# Understanding what patients think about eye care – and our services

**Published:** 2012

**Authors:** Sally Crook

**Affiliations:** Programme manager: Seeing is Believing and Consulting Editor for this issue.

**Figure:**
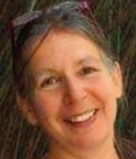
Sally Crook

All health services must be acceptable to attract patients. If patients and their families do not like the services, or the way in which they are offered, they will not use them! Without patients, our eye service will struggle, and we will not meet our VISION2020 goals.

If, like the people at Kilimanjaro Christian Medical Centre in Tanzania (see panel below), we suspect that people are not coming to us for eye services, we must try to find out why: what do they think of us and our services?

There are two main groups to consider. One group is the community as a whole. These are the potential patients, their families, and local leaders in the catchment area of the hospital or clinic. The views of the community – about eye care in general and our services in particular – will affect the overall demand for services.

The other group is those patients who have visited the eye clinic. Patients will give feedback about their experiences to their friends, neighbours, and family, and this will affect how willing others are to come forward. Finding out how these patients felt about their visit to the eye centre will help us to understand much about the community's attitude to the eye clinic. In addition, these patients can also provide helpful feedback and suggestions about how we can provide a better and friendlier service.

## Understanding the community

The best way to find out about the beliefs and practices in the local community is to visit them and talk to them. One useful way of doing so is to conduct a **knowledge, attitude, and practice (KAP) survey.**

**‘Patients can provide helpful feedback about how we can provide a better and friendlier service’**

KAP surveys are designed to find out what different people know about a topic, how they feel about it, and how they behave (their practice). For example, you could find out the following about cataract in older people:

- What do different groups of people in the community **know** about cataract? Do they know it is possible for someone who is blind from cataract to see again after a simple operation? Do they know that there is an eye clinic nearby? Do they know where it is?
- What are the **attitudes** of different groups to cataract? Do they see cataract as a normal part of growing older? Or do they see it as a health issue that must be addressed? What do they think about the eye clinic – do they trust that they will get a good outcome? Do they see it as a friendly place to go?
- What do people do about cataract? What are their practices? Would the family support an older person to come for surgery? Do people tend wait until both eyes are blind before they come for surgery? Would they come to your eye clinic, or would they prefer to travel further and go elsewhere?

**Figure:**
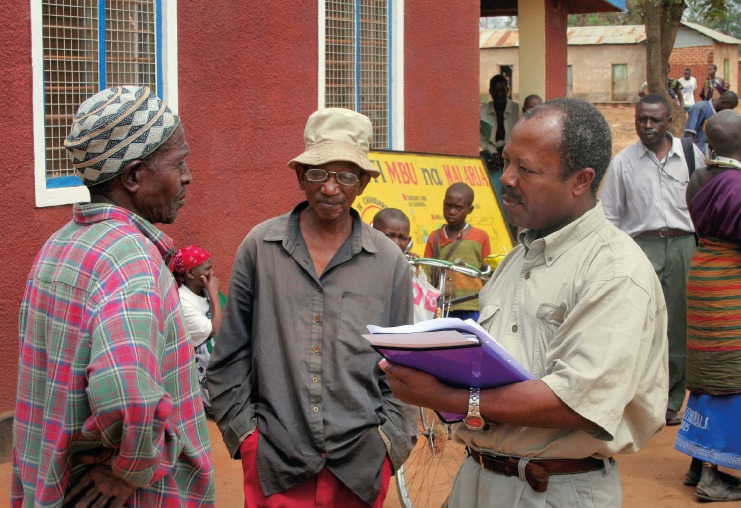
It is important to find out what patients and the community think. TANZANIA

**CASE STUDY: KILIMANJARO CHRISTIAN MEDICAL CENTRE, TANZANIA**

**A need for change at Kilimanjaro Christian Medical Centre (KCMC) in Tanzania**

A few years ago, the eye department at Kilimanjaro Christian Medical Centre (KCMC) decided to improve their cataract service. The team in charge of the improvement project discovered that, of those people blind from cataract living fewer than 50 kilometres away, only 6% were coming for cataract surgery! The team asked an ophthalmology resident to go into the community and talk to groups of pastors and village leaders. Most of the groups the resident spoke to said the same thing: people were afraid of KCMC and patients felt that they were not treated kindly.

This helped to provide the motivation to bring about considerable change at KCMC. This change involved many people and much hard work. In this issue, we have used extracts from this report to illustrate many important areas of learning.

A full report is available online from Kilimanjaro Centre for Community Ophthalmology International: www.kcco.net/Karibunibooklt.pdf (360kb). *With many thanks to the authors of the report: Susan Lewallen, Anthony Hall, John Barrows, Raheem Rahmathullah, Victoria Sheffield, Mark Swai, and John Shao*.

In this example, the KAP survey would involve talking to a range of different people, such as older people themselves, the family members who look after them, and community leaders. You would also record basic information such as gender, age, location, etc. You can then analyse the data collected to discover what key messages must be communicated to different groups in order to encourage more people to come to your eye unit.

If you are marketing your services, KAP surveys can be expanded to review local ability and willingness to pay for specific services. They can also help you assess people's understanding and use of new national health insurance schemes or new charges for in-patient services, for example.

KAP studies must be carefully designed to ensure they are as useful as possible, so it is advisable to get outside help from a local university or a non-governmental organisation with relevant experience. The World Health Organization (WHO) has much experience in designing and using KAP surveys (see ‘Further reading’, page 35).

## Understanding our patients

Day to day, it is important to know how satisfied patients are with our services and to provide a means for them to give us feedback. This can be helpful to staff as a reminder of the need to provide a good service, and managers can use this feedback as a means to motivate and encourage staff. Examples are using a complaints box (only helpful for literate patients) or giving each patient a pebble when they leave which they can put into one of two boxes: one with a ‘happy’ face and one with a ‘sad’ face.

An **exit survey** is another relatively quick and easy way to get feedback from patients as they leave the clinic. An exit survey is a series of questions that patients answer either verbally or on paper. Depending on the time and resources you have available, you can choose to get feedback from every patient or a randomly selected group of patients (every fifth patient, for example).

**Patient shadowing**

Patient shadowing is an approach that may help us understand how patients experience their journey through our eye care service.

It involves asking someone (such as an administrator or researcher) to follow a single patient, for example someone who needs cataract surgery, throughout their visit to the clinic.

Patient shadowing can help us to understand patients' physical journey through the eye service: how long each stage takes, what the waiting times are, how far they must travel within the clinic, and how easy it is for them to find their way around. This is particularly useful if we are hoping to improve patient flow (see page 31); for example, by reducing unnecessary trips or waiting times.

The person doing the shadowing can also look at the clinic or hospital environment: is it tidy, hygienic, and comfortable? Is it safe for people with visual impairment?

Finally, and most importantly, patient shadowing is an opportunity to record the patient's perceptions about the service. What is important to them? By talking to the patient, the person doing the shadowing may find out that being treated in an unfriendly way may affect the patient much more than having to wait a long time before they are seen. This will tell us where to focus our efforts to improve our eye service.

**Have you tried it?**

We would be interested to hear from you if you have tried patient shadowing. What did you learn and what were you able to change or improve as a result?

**Further reading**

- ‘Patient perspectives’ by the NHS Institute for Innovation has a detailed section on patient shadowing. **http://bit.ly/QFGRzk**
- ‘Patient shadowing’ is one of the tools on New Zealand's ‘Health service co-design’ website. www.healthcodesign.org.nz/03_explore_a.html

Exit surveys must be easy and quick to complete and be conducted in the local language. It is important to test the questions on a few patients first to make sure you are getting useful feedback.

Use volunteers, administrators, or students who rotate through your clinic to conduct the interviews. Patients will be reluctant to say anything negative to clinic staff in case it affects the care they receive in future. Take care to reassure patients that their feedback is anonymous; you may want to ask the staff conducting the survey to wear ordinary clothes or uniforms that are different from those worn by clinic staff.

Use closed-ended questions where possible: these are questions for which patients have to select a response, such as ‘yes’, ‘no’, ‘sometimes’, ‘agree’, ‘disagree’, and so on. These are easier and quicker to analyse than open-ended questions, which invite patients to answer in their own words. Using closed-ended questions is especially useful if you wish to compare responses overtime.

There is a lot of experience and helpful information available from the family planning and reproductive health sector about conducting exit interviews. For example, researchers looking at family planning clinics in Latin America found that clinics were able to make helpful changes if they were sensitive to complaints, and willing to work on issues raised by just 5% of patients (see ‘Further reading’ on page 35).

To gain a more in-depth understanding of patients’ experiences, you can also conduct individual semi-structured interviews or hold patient focus group discussions. These are can also help you to understand the community you serve.

**Time to reflect**

**Answer the following questions on a piece of paper.**

- Have you, or a family member, ever been a patient?
- How did you feel? Were you worried or scared?
- How did you want the staff to treat you or your family member?
- Did you get all the information you needed?
- Were the costs explained?
- Did you have to wait a long time without being given a reason, or without being told how much longer you would have to wait?
- What about your physical comfort: was there a comfortable seating area?
- Did you have anything to listen to or watch to help pass the time?
- Did you have access to drinking water?
- Were there toilets nearby? Were they clean and hygienic?

Read through your answers and think about your own place of work. What can you do today that will improve the experience of your patients? See pages 29-30 for additional ideas.

Write down your thoughts and discuss them with your manager or a trusted colleague. Then take action!

**Semi-structured interviews** are conducted with individuals and involve working through a list of basic questions, with the option of asking for more detail or further clarification from the person being interviewed.

**Focus groups** are similar to semi-structured interviews. A group of people who share a common characteristic (for example, people who were referred for cataract operations by the outreach programme) are chosen to take part in a discussion about a specific topic, for example how they were treated by the staff in the waiting area. A facilitator will have a list of questions to work through and will encourage the group to discuss and debate the key issues. Focus groups discussions can form the basis of further follow up and engagement with patients and the community.

For us to really understand patients’ experiences, they must feel comfortable about providing both positive and negative comments.

Again, using someone from outside the eye service to ask the questions will help people feel at ease.

Some ideas for questions are:

1. What is the best thing about your experience at our hospital/clinic today?
2. What is the worst thing about your experience here today?
3. If you could change anything about today, what would it be?

**CASE STUDY: KILIMANJARO CHRISTIAN MEDICAL CENTRE, TANZANIA**

**Making changes – and managing change**

At the eye department in Kilimanjaro Christian Medical Centre (KCMC), information about patients’ views of the service was used to convince staff that there had to be a change in the way they took care of patients. Changes were required in how they delivered services at KCMC and in how they conducted outreach.

To create these changes, it was necessary to get the support of both leaderhip and staff.

Riewing the patient pathways (patient flow – see page 31) led to changes in the length of lists in theatre and the use of key nursing staff. The team also introduced a new computerised records system. All this allowed medical, nursing, and administrative staff to clarify their roles and understand how their ways of working affected their patients.

These changes also led to improved job satisfaction from the staff themselves. According to the report, “it was heartening to see staff respond to having more concerned follow-up from supervisors, recognition and praise, the chance to use new skills in a better-organised and less frustrating environment, and the sense of camaraderie that develops when people work towards a common goal.”

This is possible thanks to good leadership and good management support.

You could ask similar questions about different aspects of the service, such as the facilities, payments/costs, the way staff treated them, how well staff explained to them what was going to happen, whether all their questions were answered clearly, their physical comfort and care (including pain management), and so on.

## Getting help

To get a more in-depth understanding of the community's beliefs and views about eye care and services, and your patients’ experience, you may want to seek outside assistance.

All methods have advantages and costs, and all require careful thought and planning. It may be advisable to ask for advice from a local university or from non-governmental organisations with experience of assisting the health sector with this work. World Health Organization offices and staff members can also offer support, including advice about what skills and expertise are available from other organisations.

